# The Living Dead: Bacterial Community Structure of a Cadaver at the Onset and End of the Bloat Stage of Decomposition

**DOI:** 10.1371/journal.pone.0077733

**Published:** 2013-10-30

**Authors:** Embriette R. Hyde, Daniel P. Haarmann, Aaron M. Lynne, Sibyl R. Bucheli, Joseph F. Petrosino

**Affiliations:** 1 Alkek Center for Metagenomics and Microbiome Research, Department of Molecular Virology and Microbiology, Baylor College of Medicine, Houston, Texas, United States of America; 2 Department of Biological Sciences, Sam Houston State University, Huntsville, Texas, United States of America; Field Museum of Natural History, United States of America

## Abstract

Human decomposition is a mosaic system with an intimate association between biotic and abiotic factors. Despite the integral role of bacteria in the decomposition process, few studies have catalogued bacterial biodiversity for terrestrial scenarios. To explore the microbiome of decomposition, two cadavers were placed at the Southeast Texas Applied Forensic Science facility and allowed to decompose under natural conditions. The bloat stage of decomposition, a stage easily identified in taphonomy and readily attributed to microbial physiology, was targeted. Each cadaver was sampled at two time points, at the onset and end of the bloat stage, from various body sites including internal locations. Bacterial samples were analyzed by pyrosequencing of the 16S rRNA gene. Our data show a shift from aerobic bacteria to anaerobic bacteria in all body sites sampled and demonstrate variation in community structure between bodies, between sample sites within a body, and between initial and end points of the bloat stage within a sample site. These data are best not viewed as points of comparison but rather additive data sets. While some species recovered are the same as those observed in culture-based studies, many are novel. Our results are preliminary and add to a larger emerging data set; a more comprehensive study is needed to further dissect the role of bacteria in human decomposition.

## Introduction

A cadaver is far from dead when viewed as an ecosystem for a suite of bacteria, insects, and fungi, many of which are obligate and documented only in such a context. Decomposition is a mosaic system with an intimate association between biotic factors (i.e., the individuality of the cadaver, intrinsic and extrinsic bacteria and other microbes, and insects) and abiotic factors (i.e., weather, climate, and humidity) and therefore a function of a specific ecological scenario. Slight alteration of the ecosystem, such as exclusion of insects or burial, may lead to a unique trajectory for decomposition and potentially anomalous results; therefore, it is critical to forensics that the interplay of these factors be understood. Bacteria are often credited as a major driving force for the process of decomposition but few studies cataloging the microbiome of decomposition have been published [1]–[5].

A body passes through several stages as decomposition progresses driven by dehydration and discernible by characteristic gross taphonomic changes. The early stages of decomposition are wet and marked by discoloration of the flesh and the onset and cessation of bacterially-induced bloat. During early decay, intrinsic bacteria begin to digest the intestines from the inside out, eventually digesting away the surrounding tissues [3]. Enzymes from within the dead cells of the cadaver also begin to break down tissues (autolysis). During putrefaction, bacteria undergo anaerobic respiration and produce gases as by-products such as hydrogen sulfide, methane, cadaverine, and putrescine [5]. The buildup of resulting gas creates pressure, inflating the cadaver, and eventually forcing fluids out [3]. This purging event marks the shift from early decomposition to late decomposition and may not be uniform; the head may purge before the trunk, for example. Purge may also last for some period of time in some parts of the body even as other parts of the body enter the most advanced stages of decomposition. In the trunk, purge is associated with an opening of the abdominal cavity to the environment [3]. At this point, the rate of decay is reported by several authors to greatly increase as larval flies remove large portions of tissues; however, mummification may also occur, thus serving to preserve tissues [6]–[9]. The final stages of decomposition last through to skeletonization and are the driest stages [7], [10]–[13].

When determining the time since death, or postmortem interval (PMI), forensic researchers may focus on progression through stages of decomposition as a function of temperature to help establish maximum and minimum time intervals for decomposition. Megyesi *et al*. [14] provide a scoring system for law enforcement agents and forensic researchers to assess decomposition as a function of time spent above a thermal minimum of physiological inactivity (accumulated degree-day). This scoring system uses bloating and purging of the head and trunk to mark the end of early decomposition. The progression of changes towards and after this point can be difficult to discern as changes are continual rather than abrupt [14]–[16]; however, bloat and purge are usually easily discernable and become a useful way to evaluate progression through stages of decomposition (personal observation).

While bloat can be an important landmark for forensic investigators for estimating roughly the postmortem interval, very little is known in the primary literature regarding the internal microorganisms involved [1]–[3], [5], [11], [17], [18]; however, numerous studies examine the microorganisms of decomposition in general and the resulting micro-environment decomposition creates [11], [17]–[25]. Much of the literature focuses on investigations of microbial activity in grave soils and have demonstrated that bacteria are useful biomarkers for forensics [1], [4], [11], [17], [18], [20]–[26]. Evans [2], Janaway [3], Vass [5], Melvin [18], and Carter [1] list a number of organisms (primarily at the familial and generic ranks though inclusive of some species) that may be significant during bloat. In common, these authors record a shift in aerobic bacteria (*Staphylococcus* and Enterobacteriacae) to anaerobic bacteria (*Clostridia* and *Bacteroides*). Janaway [3] states that the initial breakdown of tissue is due to autolysis as well as the action of bacteria present in the tissues. As the tissues breakdown, internal microorganisms (e. g. from the GI tract) spread [3]. The shift from aerobic to anaerobic organisms is likely the result of loss of redox potential of the tissue due to lack of oxygenated blood [3]. Using a mouse model, Melvin *et al*. [18] demonstrated that *Staphylococcus* species were the first microorganism to migrate from the small intestine, followed by coliform bacteria, then anaerobic bacteria. Using human cadavers, Vass [5] attempted to characterize all the microorganisms associated with decomposition and found that he was “inundated by the sheer numbers of organisms isolated” owing to the complex nature of decomposition. These studies of the microbiome of bloat are primarily based upon culture-dependent techniques and represent a gap in knowledge that needs to be addressed. It is estimated that up to 99% of bacterial species found in nature cannot be cultured by conventional means [27]. The vast majority of the microbes residing in and on the human body are difficult to culture; thus, culture-based studies are grossly incapable of accurately assessing these communities. Therefore, it is essential to use culture-independent methods to thoroughly study the relationship between humans and their microbes. Pyrosequencing of one or more variable regions of the bacterial 16S rRNA gene is a commonly used culture-independent method for analyzing membership and structure of bacterial communities. This approach enables researchers to deeply assess microbial communities collected from any environment in a high-throughput and culture-independent manner and has already been used in forensic studies analyzing the microbial communities associated with necropsy [28] and decomposition in a marine environment [20], soil [23], [29], [30], and with animal models [4].

Because bloat can be such a significant landmark in forensics indicating the end of the early stages of decomposition and the shift to the late stages, and so little is known regarding the bacterial basis of bloat, we conduct one of the first exploratory investigations into the internal microbiome of cadavers placed in an outdoor environment to decompose under natural conditions. The majority of body sites sampled in our study have not been sampled from living human beings as such sampling would be invasive, painful, or impossible. These data are the first of their kind and represent the initial cataloguing of bacterial species associated with the bloat stage of human decomposition by targeting time points at the onset and end of the bloat stage.

## Methods

### Placement of cadavers

This research used cadavers donated to the Southeast Texas Applied Forensic Science (STAFS) Facility at the Center for Biological Field Studies (CBFS) at Sam Houston State University (SHSU), which is a willed body donation facility. All donors or next of kin sign a donation form that relinquishes all rights and claims regarding the body after death, making it available for teaching or scientific purposes. Within the form, it is made clear that the cadavers will be placed outside to decompose under natural conditions and various samples (bacteria, tissue, insect, etc.) will be taken for use in appropriate research studies. Upon request, families and/or next of kin are debriefed on research or educational projects for which their donation may be used. Researchers at STAFS sign a confidentiality form waiving access to any personal and identifying information about the cadavers. The SHSU Institutional Review Board, the Protection of Human Subjects Committee, has determined that IRB approval is not required for use of human cadavers since no personal or identifiable information is collected.

The CBFS and STAFS facility within is approximately 5 km north of Huntsville, Texas (Walker County) and is characterized by a large distribution of pine trees, acidic soils, and a humid, subtropical climate [31]. The STAFS human decomposition facility is a two-acre area fenced off within the larger 247 acres of CBFS and that has a sparse forest covering of pine trees and a ground covering of herbaceous plants.

For this experiment, two cadavers were placed sequentially in this outdoor facility to decompose under natural conditions. STAFS 2011-006 was sampled from 8 – 15 September 2011 and STAFS 2011-016 was sampled from 3 – 17 November 2011. Due to the nature of the donation program, it is difficult to acquire cadavers that are similar in physical, medical, cause of death, or postmortem conditions, but efforts to reduce variables are made to the best of our abilities. Neither cadaver had been autopsied and each was assessed initially according to the Megyesi *et al*. [14]body scoring system to be in early stages of decomposition, i.e., did not show visible signs of the bloat process. Each was a Caucasian male and placed supine without clothing. STAFS 2011-016 was 68 years old, with a medical history of diabetes mellitus, chronic alcoholism, and cardiovascular disease with acute myocardial infarction as the cause of death. The time since death is unknown with 89 days spent frozen (−17 to −12oC) and 13 days spent in the cooler (3.33oC). STAFS 2011-006 was 52 years old with no medical history beyond carbon monoxide poisoning as the cause of death. The time since death is unknown with 143 days spent frozen (−17 to −12oC) and 6 days spent in the cooler (3.33oC). For both cadavers, the exact height and weight are unknown but were estimated to be approximately average height and slightly above average weight for current U.S. standards [32].

**Figure 1:**
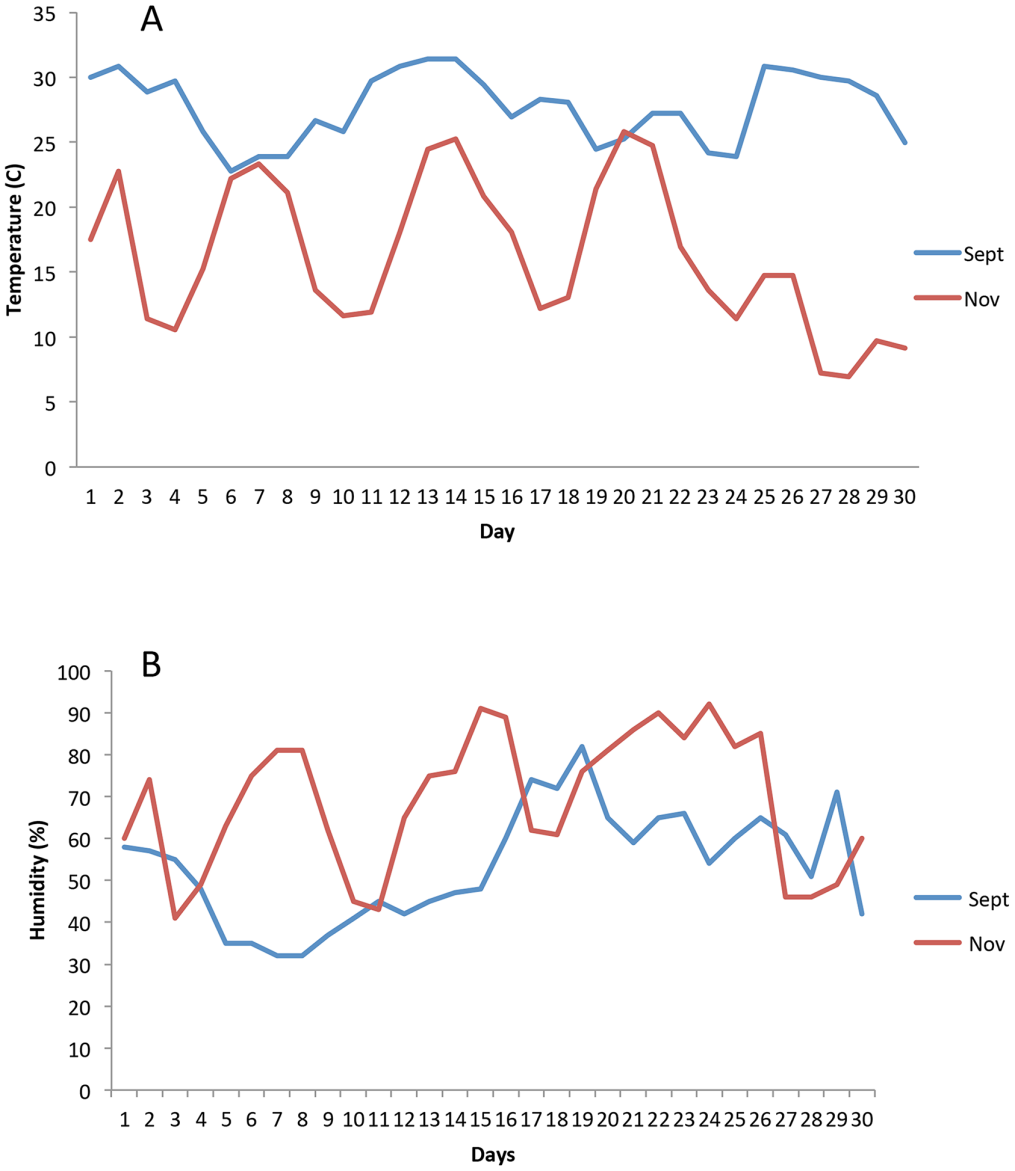
(A) and humidity (B) for September 2011 (sampling of STAFS 2011-006) and November 2011 (sampling of STAFS 2011-016).

**Figure 2:**
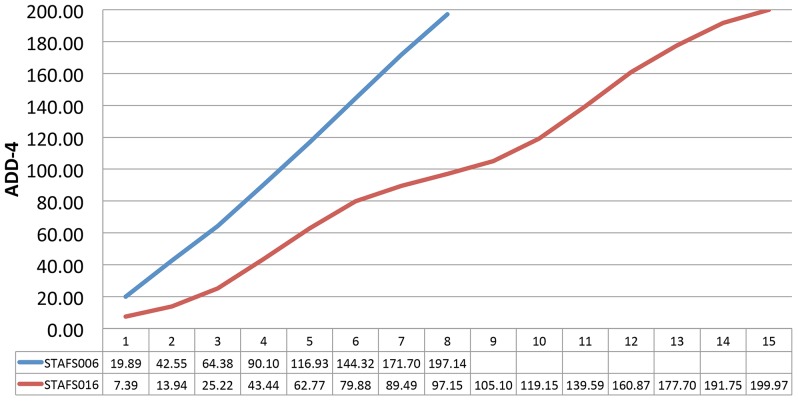
Accumulated degree hours calculated at a base of 4oC for STAFS 2011-006 and STAFS 2011-016. Bodies were samples at the start of decomposition and at a point determined to be almost the end of the bloat stage. DD =  ((Maximum Temperature + Minimum Temperature)/2)-base temperature; ADD =  (DDx+DDx+1).

Average daily temperature and humidity conditions for September and November 2011 were calculated using data from National Oceanic and Atmospheric Administration (http://www.ncdc.noaa.gov) (Figure 1). Accumulated degree-days above a base of 4oC (Figure 2) for each cadaver were calculated using the method of Micozzi *et al*. [12].

### Collection of bacterial samples

To assess bacterial taxa present during decomposition, samples were taken from two time points: at placement (referred to as pre-bloat for simplicity) and conclusion (referred to as end-bloat for simplicity) (Table 1). Pre-bloat samples were taken from mouth and rectum only to avoid the introduction of artificial wounds. Mouth “swabs” were samples taken by swabbing the inside of the mouth with a sterile cotton applicator. The tip of the applicator was cut and placed in a collection tube of sterile phosphate buffered saline (PBS). Mouth and rectal “scrapings” were samples taken by scraping the inside of the mouth or rectum using a sterile plastic disposable spatula. The tip of the disposable spatula was also cut and stored in sterile PBS. The mouth of each cadaver was slightly open when each sample was collected. End-bloat sampling was targeted to occur as close as possible to the end of the bloat stage, which is marked by purging of decomposition fluids, but before purge. The body scoring system of Megyesi *et al*. [14] for the trunk was used to determine this sample time. The point within category B (early decomposition) between stage 2 (gray to green discoloration: some flesh relatively fresh) and stage 3 (bloating with green discoloration and purging of decompositional fluids), but closer to stage 3 than to stage 2, was targeted. For the end-bloat sample, maggots (common at this stage of decomposition) were removed using a sterile plastic disposable spatula prior to sampling. To access the internal organs at end-bloat sample time, each cadaver was dissected through the abdomen. Since this procedure was destructive, it marked the end of sampling, and resulted in a non-identical sampling regime.

**Table 1:** 

Date	STAFS 2011-006	Date	STAFS 2011-016
8-Sep-11	Pre-bloat	3-Nov-11	Pre-bloat
	Mouth swab		Mouth swab
	Mouth scrape		Mouth scrape
	Rectal scrape		Rectal scrape
15-Sep-11	End-bloat	17-Nov-11	End-bloat
	Mouth scrape		Mouth scrape
	Small intestine swab		Small intestine swab
	Transverse colon swab		Transverse colon swab
	Sigmoidal colon swab		Stomach scrape
	General body cavity swab		

### Sample processing and 454 pyrosequencing

Sample processing, 16S rRNA gene amplification, and 454 pyrosequencing was performed following protocols benchmarked as part of the Human Microbiome Project [33], [34]. Bacterial genomic DNA was extracted from samples using the PowerSoil DNA Isolation Kit (MoBio, Carlsbad, CA). The V3-V5 regions of the 16S rRNA gene were amplified from genomic DNA using primer 357F (5′-CCTACGGGAGGCAGCAG-3′) and barcoded primer 926R (5′-CCGTCAATTCMTTTRAGT-3′). Both primers were modified with the addition of 454 FLX-titanium adaptor sequences. 10 µL DNA and 2 µL of 4 µM paired primer stock was used in a 20 µL PCR reaction with 0.15 µL AccuPrime High Fidelity *Taq* polymerase and 5.85 µL PCR water. Reactions were heated for 2 minutes at 95oC followed by 30 cycles of 20 seconds at 95oC, 45 seconds at 50oC, and 5 minutes at 72oC. Amplicons were sequenced on a multiplexed 454-FLX-Titanium pyrosequencing run at the Human Genome Sequencing center at Baylor College of Medicine. Raw sequence data are deposited in the Sequence Read Archive (BioSample accession SAMN02358582-SAMN02358599).

### Data analysis with QIIME

16S data were processed and analyzed using QIIME version 1.7.0 [26], an open-source software package. The sequence file was demultiplexed and reads were quality filtered and trimmed. All sequences outside of the minimum (200 nucleotides) and maximum (1000 nucleotides) length bounds, with any ambiguous bases, with a homopolymer longer than 6 nucleotides, more than two primer mismatches, and more than one barcode mismatch were removed. Additionally, an average quality score of 30 over a 50 base pair sliding quality window was required; sequences were trimmed at the first base of the low quality score window and any sequences less than 200 nucleotides in length after truncation were removed. Quality trimming resulted in 114,650 high quality reads. Two samples, body cavity sample STAFS 2011-006 and body cavity sample STAFS 2011-016, did not have any reads, thus, we could not include these samples in analysis. Sequences were binned into operational taxonomic units (OTUs) using uclust with a sequence identity of 97%, and singleton OTUs (those OTUs with only one read), accounting for 1.4% of reads, were removed from the dataset (Table S1). Taxonomy was assigned using RDP Classifier (version 2.2) trained to the greengenes database (October 2012 release). The OTU table was subsampled so that each sample had 1747 reads associated with it (the smallest number of reads associated with any one sample), and this standardized OTU table was used for alpha and beta diversity analyses. Samples were grouped according to body site, and further sub-grouped according to sample collection method (swab or scrape), bloat stage (pre-bloat or end-bloat), and cadaver of origin (STAFS 2011-006 or STAFS 2011-016) for these analyses.

## Results and Discussion

Temperatures during September 2011 were warmer on average than in November 2011 with lower relative humidity levels (Figure 1). STAFS 2011-016 took longer to decompose and stayed wet longer, likely due to the higher relative humidity and cooler temperatures of November 2011. Although decomposition progresses in a predictable manner, the duration of the stages of decomposition are variable and it is not obvious when a cadaver will experience purge. The time for end-bloat sampling was determined using the qualitative aspect of the scoring system presented by Megyesi *et al*. [14] which resulted in non-identical intervals between sampling events between each cadaver. However, it is also common to assess decomposition quantitatively via accumulated degree-days. Accumulated degree-day illustrates decomposition as a temperature-based physiological process rather than a time-based calendar process and measures the amount of time spent above a thermal minimum threshold (recorded as heat-energy units) at which physiological activity of the cadaver and bacteria (and insects) is slowed or stopped [14], [16], [35]. Accumulated degree-days were calculated *post-hoc* to determine the difference in physiological time accumulated between the two cadavers. Using a base of 4oC the cadavers varied by 2.83 heat-energy units at the end of the study: STAFS 2011-006 accumulated 197.14 heat-energy units while STAFS 2011-016 accumulated 199.97 heat-energy units when end-bloat samples were taken (Figure 2). The thermal minimum of 4oC was chosen for this comparison with the assumption that the internal microorganisms are mesophiles and metabolism is inhibited at this temperature [12]. While we used a thermal base of 4oC, it is important to note that not all researchers in the field agree on an appropriate thermal minimum to use for the ADD calculations. Michaud and Moreau [15] uses a base of 5oC, Micozzi [12] uses a base of 4oC, Megyesi *et al*. [14] use a base of 0oC, and Myburgh *et al*. [16] use only daily averages as their study site never falls below a thermal minimum. If a lower thermal minimum such as 0oC is used, STAFS 2011-016 would accumulate more thermal heat-energy units than STAFS 2011-006 (by about 30). It is not known at which temperature decomposition ceases as experimental evidence for the appropriate thermal minimum is lacking [14]. Therefore, for this research, we thought it most meaningful to rely on the total body scoring system of Megyesi *et al*. [14] to determine the time for end-bloat sampling.

The variable conditions of decomposition surrounding each cadaver could greatly influence diversity of intrinsic and extrinsic bacterial communities and therefore could have an impact on the overall process of decay. Many factors can influence the bacteria detected in and on a cadaver, including the individual's “starting” microbiome, differences in the decomposition environments of the two cadavers, and differences in the sites sampled at end-bloat. The integrity of organs at end-bloat varied between cadavers (as decomposition varied between cadavers) and did not allow for consistent sampling of sites across cadavers. Specifically, STAFS 2011-016 no longer had a sigmoidal colon at the end-bloat sample time. While this could skew a comparison analysis, it provided us with a broad overview of the microbiome of decomposition. For this reason, the most meaningful approach is to view the data as complimentary, not comparatively, and as contributing to a larger emerging additive data set (STAFS 2011-006 plus STAFS 2011-016 rather than STAFS 2011-006 versus STAFS 2011-016); however, some within body comparisons can be made (pre-bloat samples versus end-bloat samples of STAFS 2011-006; pre-bloat samples versus end-bloat samples of STAFS 2011-016).

**Figure 3:**
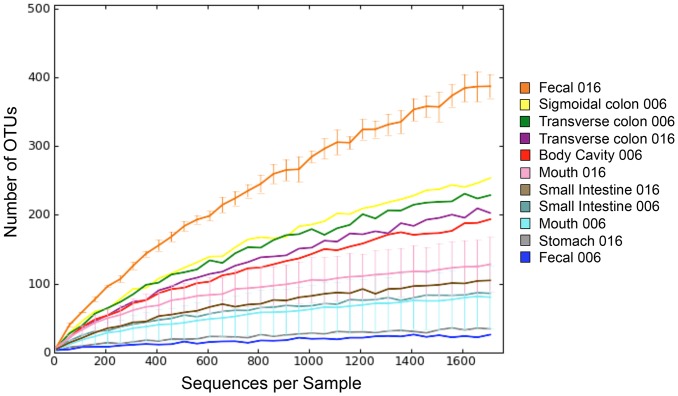
Microbiome richness varies widely across cadaver body sites. A rarefaction curve plotting the average number of OTUs over the average number of sequences sampled per body site sampled shows that microbiome richness differs widely among different body site samples. Error bars represent the standard error of the mean. The legend indicates the body site and body identification number for each sample.

We first surveyed the richness of the microbial communities sampled from each cadaver body site. The mouth and stool of the living have been sampled extensively in various microbiome projects, most notably the Human Microbiome Project (HMP) [33], [34]. The HMP found that stool harbors a rich microbiome while the oral cavity is much less rich [33]; we observed the same pattern in samples collected from the cadavers (Figure 3). It should be noted, however, that the cadavers do not fall into the HMP age range or acceptable medical history [33], [34], and because of this there is a limitation to how directly these two data sets can be compared. With the exception of the fecal sample from STAFS 2011-006, which was the least rich sample in the study with only 26 unique OTUs detected, fecal samples were the richest of all body sites sampled, with an average of nearly 400 OTUs detected. The stomach sample was the second least rich sample, with small intestine and mouth samples slightly richer. The body cavity, transverse colon, and sigmoidal colon samples were much richer. Overall, these data show that as one moves from the upper gastrointestinal tract (mouth, stomach, and small intestine) to the lower gastrointestinal tract (colon and rectal/fecal), microbiome richness increases.

**Figure 4:**
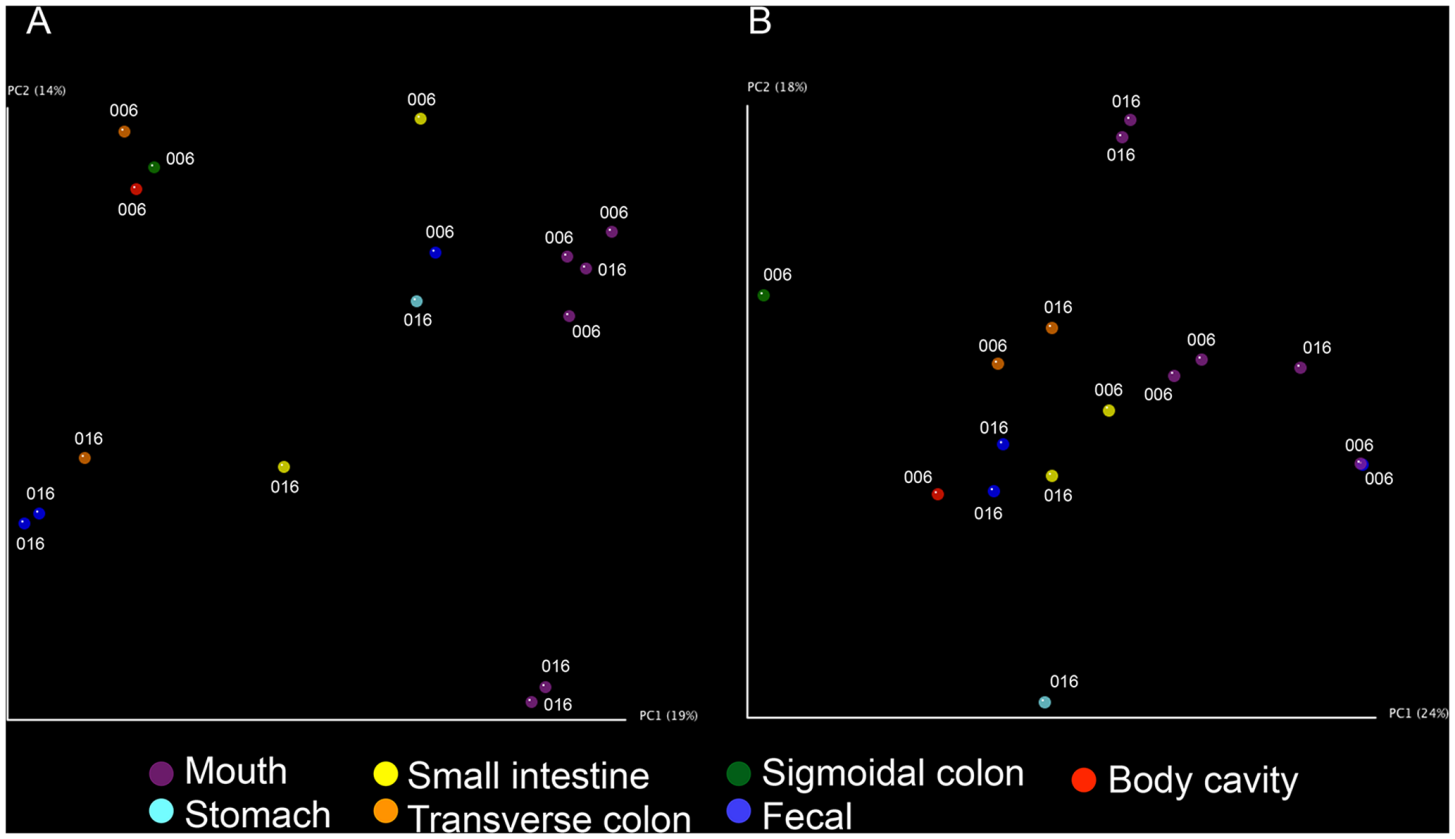
Principal coordinate analysis reveals separation of communities based on location in the gastrointestinal tract. The first two principal coordinates (PC) of Unweighted (A) and Weighted (B) UniFrac-based principal coordinate analysis are plotted, and the percent variance explained by each PC is indicated in parenthesis.

We next analyzed beta diversity (diversity between samples) through UniFrac-based Principal Coordinates Analysis (PCoA), which revealed differences in microbiome composition and structure across body sites. UniFrac is a phylogenetic-tree based distance metric that is calculated as the percent of branch length leading to descendants from one sample or the other [36]. Weighted UniFrac additionally takes into account the relative abundances of sequences present in each sample, which contribute weight to the branches of the tree. Samples that share more branch length have more similar microbial communities and when plotted onto a PCoA plot cluster near one another. On both Unweighted and Weighted UniFrac-based PCoA plots, we observed a right to left movement on PC1 as samples went from the upper to the lower gastrointestinal tract (Figure 4). The separation between samples based on location in the gastrointestinal tract was more notable on the Unweighted UniFrac-based plot. On both plots, a fecal sample from STAFS 2011-006 clustered very near a mouth sample from the same body. This sample was also the least rich sample (Figure 3), and stool samples are typically the richest microbiome samples [33], [34], thus, it appears that this sample is not a typical fecal sample. This may be due to sampling error, poor sample quality, or some condition unique to the cadaver. The remainder of the samples show stark differences between oral and fecal samples with mouth and fecal samples each forming separate clusters. This clustering pattern is not surprising as other studies have shown that oral communities differ from GI tract communities [33], [34], [37], [38]. The unique opportunity to sample the general body cavity was also presented in this study. While only one body cavity sample produced any reads, it is interesting to note that it clustered closely with large intestine samples. The source of the bacterial communities in the body cavity is unknown, but it is possible that bacteria could have migrated out of the large intestine as it started to decompose. Previous studies provide support for this hypothesis [18]. Another possible source of these communities may be external body locations (e.g. skin) or environmental (e.g. soil) bacteria. No conclusions can be drawn regarding these communities as these samples were not collected during this study. Any subsequent studies that wish to address the source of any newly colonizing communities should include these sample sets.

**Figure 5:**
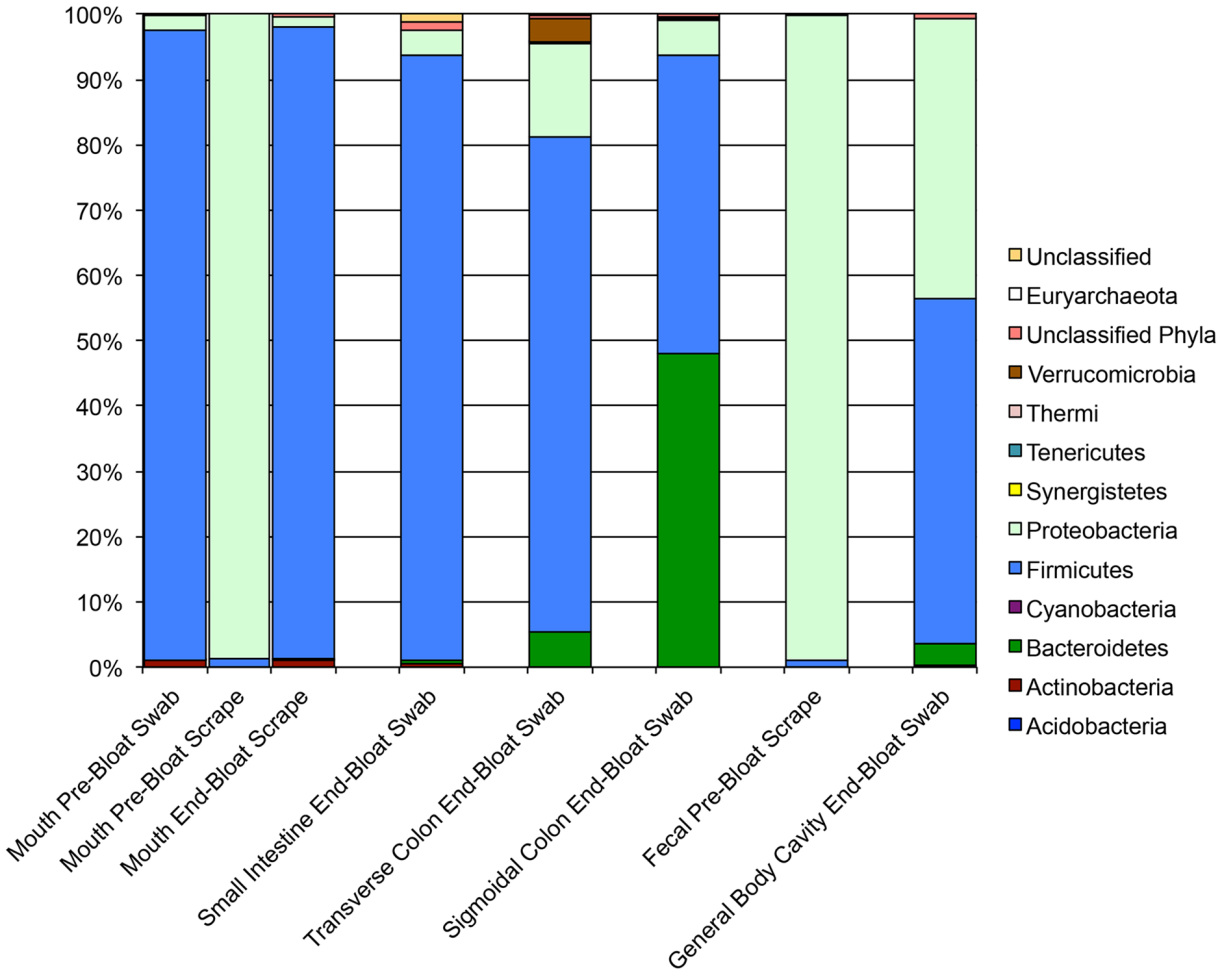
The relative abundances of phyla present differ between sample sites in STAFS 2011-006. Stacked bar charts illustrate the relative abundances of phyla present from each individual sample collected from STAFS 2011-006.

**Figure 6:**
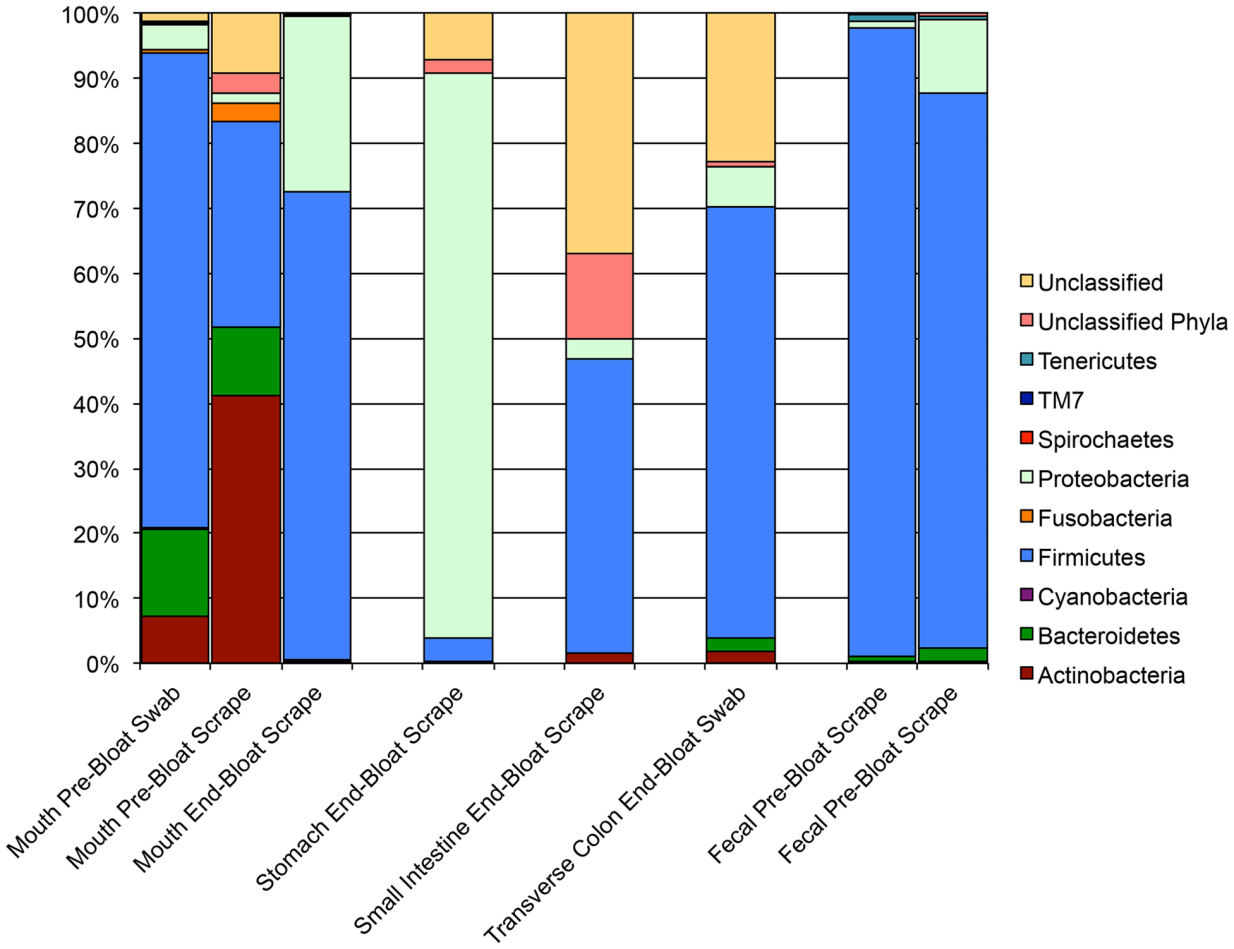
The relative abundances of phyla present differ between sample sites in STAFS 2011-016. Stacked bar charts illustrate the relative abundances of phyla present from each individual sample collected from STAFS 2011-016.

As another measure of beta diversity, we analyzed the relative abundances of phyla present in each group of samples. As Figures 5 and 6 show, the microbiome composition differed notably across body sites with the most prevalent phylum being the Firmicutes. Proteobacteria were also present at an appreciable abundance in most samples. Notably, there were very few unclassified reads associated with STAFS 2011-006, but there were an appreciable amount of unclassified reads associated with STAFS 2011-016, particularly in the small intestine sample. Firmicutes mainly dominated the two fecal samples from STAFS 2011-016 with very few Bacteroidetes detected. In healthy humans, the two most abundant phyla in feces are Firmicutes and Bacteroidetes, with a ratio ranging from 1∶10 to 10∶1 [39], thus, the pre-bloat fecal samples from STAS 2011-016 do not differ significantly from what might be expected in a living person. In contrast to STAFS 2011-016, the fecal sample from STAFS 2011-006 was dominated by Proteobacteria. This sample was the sample that was discussed above due to its low richness and clustering with an oral sample (the Proteobacteria dominated oral sample from STAFS 2011-006, Figure 5). An increase in Proteobacteria has been associated with certain gastrointestinal disorders [40], [41], thus, it is possible that this increase is due to an unknown disease state of the subject before death. Alternatively, these results may be due simply to sample contamination.

Although we saw notable differences in the relative abundances of phyla present between cadaver body sites, we desired a more in-depth survey of the bacterial communities; thus, we determined the relative abundances of genera present in each body site for each cadaver. As with the data collected at the phylum level, we saw stark differences in the bacterial genera detected between bodies and between body sites within the same body. Supplementary tables 2 and 3 list the top ten genera from each body site sampled from each cadaver. Samples from the lower gastrointestinal tract and body cavity of STAFS 2011-006 (transverse and sigmoidal colon) share a number of genera, most notably *Clostridium, Lactobacillus, Eggerthella*, and *Bacteroides*. In contrast, in both bodies, samples from the upper gastrointestinal tract (mouth, stomach, and small intestines) are quite different in composition from those from the lower gastrointestinal tract. Interestingly, the pre-bloat mouth samples from STAFS 2011-016 more closely replicated what is seen in a “healthy” human oral cavity than did the mouth samples from STAFS 2011-006, with *Streptococcus, Prevotella, and Veillonella* detected among the top ten genera in the both the pre-bloat swab and scrape (only *Streptococcus* was detected among the top ten genera in the pre-bloat swab and scrape from STAFS 2011-006, compare supplementary tables 1 and 2). These three genera are among the top five genera detected in the oral cavity in the HMP Cohort [33], [34].

We analyzed the relative abundances of taxa present in oral samples collected pre-bloat and end-bloat to determine whether and how bacterial communities vary at different stages of decomposition. The mouth was the only sample site for which we had both pre- and end-bloat samples. We could not sample the small intestines, transverse colon, and sigmoidal colon pre-bloat as sampling these sites initially would have compromised the integrity of the body wall and altered decomposition, and we could only sample the feces pre-bloat, as the rectum no longer existed at the end-bloat sample time. Two pre-bloat mouth samples were taken from STAFS 2011-006: swab and scrape. The bacterial communities of these two samples varied greatly. The pre-bloat swab was predominated by Firmicutes while the pre-bloat scrape was predominated by Proteobacteria. Only one end-bloat mouth scrape was very similar to the pre-bloat mouth swab in that it was predominated by Firmicutes (Figure 5). For STAFS 2011-016, two pre-bloat samples were also taken: swab and scrap. While the two samples varied in relative abundance, they were more similar to each other than they were to STAFS 2011-006. Both samples had a significant percentage of Firmicutes, Bacteriodetes, and Actinobacteria, though the pre-bloat swab was predominated by Firmicutes while the pre-bloat scrape was predominated by Actinobacteria. The STAFS 2011-016 end-bloat scrape was predominated by Firmicutes followed by Proteobacteria (Figure 6). Based upon these results, it appears that sample collection methodology has an effect on the detected relative abundance. Supplementary tables 2 and 3 show very little overlap in similarity at the genus level (or more inclusive levels) within sites of the same bodies. While the goal of this study was not to determine the best methodology for sample collection, a study to determine the best method may be warranted.

Previous decomposition studies have recorded a shift from communities dominated by aerobic bacteria (*Staphylococcus* and Enterobacteriacae) to those dominated by anaerobic bacteria (*Clostridia* and *Bacteroides*). Our data support these findings and add to them by recovering previously undocumented bacteria. Traditional culture methods have implicated particular bacteria present during human decomposition [3], [5], [29]. While very useful, traditional methods may not yield an accurate account of species diversity as they can detect only those species that can be grown in culture (based on molecular studies, it is estimated that perhaps only 1% of bacteria can be cultured [27]). Culture methods suggest that the obligate anaerobes, *Clostridium* and *Bifidobacterium* in particular, are the most abundant microbial species observed during putrefaction within the GI tract, with smaller amounts of facultative anaerobic bacteria present (such as *Lactobacillus* and members of Enterobacteriaceae). Our results indicate that *Clostridium* is abundant at the end of the bloat stage in most of the GI tract locations that were sampled: the small intestine, transverse colon, and sigmoidal colon, but not the stomach. *Clostridium* was found in relatively high abundance (up to 60% in the small intestine) but not at the reported level of 90% by other researches [3]. *Bifidobacterium* was among the top ten genera detected in only the transverse colon end-bloat sample from STAFS 2011-016, and at a low relative abundance of 1.71%. *Lactobacillus* was relatively abundant in all GI tract samples from STAFS 2011-006, ranging in abundance from 5% to 29%. One member of Enterobacteriaceae, *Escherichia*, was detected in the lower GI tract samples in both bodies, for both pre-bloat and end-bloat.

*Clostridium* species are thought to progress decomposition by breaking down lipids and complex carbohydrates associated with human tissue [3]. The lipases of *Clostridium* are thought to significantly aid the hydrolysis of fat under warm and moist conditions, while hydrolytic enzymes will convert carbohydrates to organic acids and alcohols [3]. Bacteria such as *Pseudomonas, Bacillus*, and other sulphate-reducing bacteria of the GI tract are often proteolyic and have been reported to be important in the breakdown of proteins of a cadaver [3]. *Pseudomonas* was detected in pre-bloat samples from STAFS 2011-006 (mouth scrape and fecal scrape) but was not detected in any end-bloat sample (supplementary tables 2 and 3). In both the mouth and GI tract end-bloat samples, *Pseudomonas* was not among the top ten genera detected, being replaced by other common GI tract bacteria (*Clostridia, Lactobacillus*, etc.). This shift is likely due to the loss of redox potential (at least in the GI tract) and would likely be responsible for the breakdown of tissue proteins. It is important to note that while difference in abundance seen in particular species between this study and the others noted above could be due to the discussed constraints of culturing bacteria, differences could also be due to a variety of factors such as individual variability between the cadaver microbiomes, seasonality, climate, and species of colonizing insects. Finally, abundance does not necessarily indicate metabolic significance for decomposition, a point of importance that our study cannot address.

In studies using cadavers as research subjects, a realistic concern centers on small sample sizes and lack of statistical significance. Cadavers for decomposition research are difficult to obtain, can be a limited resource, require special handling, can be expensive, and are nearly impossible to set up in replicate. A reasonable way around these issues is to use animal carcass models rather than human cadavers [42] with pigs as a preferred model, especially in entomological studies, due to similarities with humans in terms of diet preference, body fat, and hair coverage [43]. However, studies of the pig microbiome have demonstrated that the bacterial communities of pigs and humans are not comparable [44], with pig feces having bacterial phyla most similar to that of the cow rumen and chicken cecum [45]. Additionally, tests of the validity of using animal models in soil decomposition studies of skeletal muscle have shown that there are no precise non-human tissue predictors for human tissues [42]. Therefore, to more accurately assess the microbiome associated with bloat, we chose to take advantage of our unique research facilities at STAFS and use human cadavers as test subjects, recognizing that the data sets from each body are not comparable due to low sample size, individual variation, and sequential placement. Even though we have a limited sample set, as additional data are collected from subsequent bodies, this will allow for increased comparisons and enable a more thorough characterization of decomposition.

## Conclusions

This analysis is largely exploratory and provides the first catalogue of bacteria present internally for the onset and end of the bloat stage of decomposition. Our data represent initial insights into the bacteria populating decomposing human cadavers and an early start to discovering successive changes through time. While our data support the findings of previous culture studies, they also demonstrate that bacteria not detected by culture-based methods comprise a large portion of the community. No definitive conclusion regarding a shift in community structure through time can be made with this data set. To be able to establish a successive change, more cadavers should be tested with more sample time points and more sample locations. Future studies should investigate the change in bacterial community structure as a function of time in a longitudinal survey that will extend beyond bloat to skeletal remains. Other useful studies will investigate extrinsic sources of bacteria brought by insects and the soil. Ultimately, to accomplish the goal of understanding decomposition as a mosaic ecosystem, holistic investigations uniting the disparate fields of microbiology, entomology, and chemistry should be done.

## Supporting Information

Table S1 **The number of sequences associated with each sample (listed as sample ID with body site and body number in parenthesis) post-trimming and after removing singleton reads is listed. 1.4% of reads were singletons.**
(DOCX)
