# Supplementary material for: The Living Dead: Bacterial Community Structure of a Cadaver at the Onset and End of the Bloat Stage of Decomposition
Source: PLoS One. 2013 Oct 30;8(10):e77733. doi: 10.1371/journal.pone.0077733 (PMC3813760; doi:10.1371/journal.pone.0077733)
Supplement: Table S1 — The number of sequences associated with each sample (listed as sample ID with body site and body number in parenthesis) post-trimming and after removing singleton reads is listed. 1.4% of reads were singletons. (DOCX) [file pone.0077733.s001.docx]

| **Table S1** | | |
| --- | --- | --- |
| Sample ID (Sample Site and Body Number) | Number of Sequences Post-Trimming | Number of Sequences After Singleton Removal |
| Cad1 (Mouth 006) | 7814 | 7762 |
| Cad2 (Sm. Intestine 006) | 4503 | 4492 |
| Cad3 (Transverse Colon 006) | 11046 | 10897 |
| Cad4 (Body Cavity 006) | 0 | 0 |
| Cad5 (Body Cavity 006) | 8019 | 7960 |
| Cad6 (Mouth 016) | 10146 | 10060 |
| Cad7 (Body Cavity 016) | 0 | 0 |
| Cad8 (Transverse Colon 016) | 10163 | 9997 |
| Cad9 (Mouth 006) | 8266 | 8258 |
| Cad10 (Fecal 006) | 6565 | 6550 |
| Cad11 (Mouth 006) | 5567 | 5505 |
| Cad12 (Sigmoidal Colon 006) | 10634 | 10433 |
| Cad13 (Mouth 016) | 7392 | 7308 |
| Cad14 (Fecal 016) | 8126 | 7732 |
| Cad15 (Fecal 016) | 7483 | 7205 |
| Cad16 (Stomach 016) | 3199 | 3184 |
| Cad17 (Mouth 016) | 3957 | 3933 |
| Cad18 (Sm. Intestine 016) | 1770 | 1747 |

| **STAFS 2011- 006** | | | |
| --- | --- | --- | --- |
| **Mouth pre-bloat swab** | | **Mouth pre-bloat scrape** | |
| Genus | Abundance | Genus | Abundance |
| Staphylococcus (Firmicutes) | 57.0% | Pseudomonas (Proteobacteria) | 90.6% |
| Peptoniphilus (Firmicutes) | 18.8% | Unclassified, Family Pseudomonadaceae | 6.36% |
| Clostridium (Firmicutes) | 8.83% | Unclassified Genus, Class Gammaproteobacteria | 1.62% |
| Lactobacillus (Firmicutes) | 5.40% | Lactobacillus (Firmicutes) | 0.560% |
| Ignatzschineria (Proteobacteria) | 1.37% | Atopobium (Firmicutes) | 0.411% |
| Unclassified, Family Staphylococcaceae | 1.06% | Streptococcus (Firmicutes) | 0.230% |
| Unclassified, Class Bacilli | 1.04% | Propionibacterium (Actinobacteria) | 0.048% |
| Streptococcus (Firmicutes) | 0.618% | Unclassified, Family Clostridiaceae | 0.048% |
| Unclassified, Family Peptostreptococcaceae | 0.570% | Sphingomonas (Proteobacteria) | 0.048% |
| Unclassified, Family Carnobacteriaceae | 0.490% | Unclassified, Family Bacillaceae | 0.024% |
| **Mouth end bloat scrape** | | **Small intestine end bloat swab** | |
| Peptoniphilus (Firmicutes) | 41.9% | Clostridium (Firmicutes) | 59.8% |
| Clostridium (Firmicutes) | 22.5% | Lactobacillus (Firmicutes) | 28.9% |
| Unclassified, Family Clostridiaceae | 12.1% | Sphingomonas (Proteobacteria) | 1.49% |
| Lactobacillus (Firmicutes) | 4.70% | Unclassified | 1.27% |
| Staphylococcus (Firmicutes) | 4.58% | Unclassified, Family Comamonadaceae | 1.18% |
| Unclassified, Family Clostridiaceae | 4.32% | Unclassified, Family Peptostreptococcaceae | 1.16% |
| Unclassified, Family Carnobacteriaceae | 1.74% | Unclassified Bacteria | 1.14% |
| Peptostreptococcus (Firmicutes) | 1.18% | Unclassified, Family Clostridiaceae | 0.801% |
| Unclassified, Family Peptostreptococcaceae | 0.944% | Eggerthella (Firmicutes) | 0.712% |
| Sphingomonas (Proteobacteria) | 0.727% | Unclassified, Order Rickettsiales | 0.490% |
| **Transverse colon swab** | | **Sigmoidal colon swab** | |
| Lactobacillus (Firmicutes) | 25.3% | Bacteroides (Bacteroidetes) | 42.0% |
| Clostridium (Firmicutes) | 20.1% | Clostridium (Firmicutes) | 17.3% |
| Unclassified, Family Peptostreptococcaceae | 11.8% | Lactobacillus (Firmicutes) | 5.12% |
| Escherichia (Proteobacteria) | 11.7% | Unclassified, Family Rikenellaceae | 4.51% |
| Eggerthella (Firmicutes) | 7.75% | Escherichia (Proteobacteria) | 3.98% |
| Bacteroides (Bacteroidetes) | 4.26% | Unclassified, Family Ruminococcaceae | 3.43% |
| Akkermansia (Verrucomicrobia) | 3.62% | Eggerthella (Firmicutes) | 2.96% |
| Unclassified, Family Enterobacteriaceae | 2.61% | Eubacterium (Firmicutes) | 2.16% |
| Unclassified, Family Lachnospiraceae | 1.87% | Unclassified, Family Peptostreptococcaceae | 1.85% |
| Eubacterium (Firmicutes) | 1.10% | Unclassified, Family Lachnospiraceae | 1.29% |
| **Fecal scrape** | | **Body cavity end bloat swab** | |
| Pseudomonas (Proteobacteria) | 85.0% | Escherichia (Proteobacteria) | 24.6% |
| Unclassified, Family Pseudomonadaceae | 7.98% | Eubacterium (Firmicutes) | 13.2% |
| Psychrobacter (Proteobacteria) | 3.11% | Erwinia (Proteobacteria) | 13.1% |
| Unclassified, Class Gammaproteobacteria | 2.0% | Clostridium (Firmicutes) | 10.9% |
| Unclassified, Family Moraxellaceae | 0.687% | Lactobacillus (Firmicutes) | 6.11% |
| Dorea (Firmicutes) | 0.153% | Collinsella (Firmicutes) | 5.98% |
| Peptoniphilus (Firmicutes) | 0.137% | Ruminococcus (Firmicutes) | 5.87% |
| Bacteroides (Bacteroidetes) | 0.122% | Unclassified Genus, Family Enterobacteriaceae | 4.75% |
| Streptococcus (Firmicutes) | 0.122% | Bacteroides (Bacteroidetes) | 2.90% |
| Unclassified Bacteria | 0.076% | Eggerthella (Firmicutes) | 1.68% |

**Supplementary Table 2:** Relative abundances of the top ten genera present in each sample from body 006.

| **STAFS 2011- 016** | | | |
| --- | --- | --- | --- |
| **Mouth pre-bloat swab** | | **Mouth pre-bloat scrape** | |
| Genus | Abundance | Genus | Abundance |
| Streptococcus (Firmicutes) | 32.5% | Microbacterium (Actinobacteria) | 35.1% |
| Prevotella (Bacteroidetes) | 11.2% | Streptococcus (Firmicutes) | 13.8% |
| Veillonella (Firmicutes) | 9.74% | Prevotella (Bacteroidetes) | 9.34% |
| Unclassified, Family Gemellaceae | 5.83% | Unclassified | 9.21% |
| Atopobium (Firmicutes) | 4.50% | Veillonella (Firmicutes) | 5.61% |
| Unclassified, Family Clostridiaceae | 3.96% | Unclassified, Order Actinomycetales | 3.71% |
| Unclassified, Family Carnobacteriaceae | 2.78% | Unclassified Bacteria | 2.94% |
| Megasphaera (Firmicutes) | 2.71% | Megasphaera (Firmicutes) | 1.79% |
| Dialister (Firmicutes) | 1.88% | Fusobacterium (Fusobacteria) | 1.63% |
| Actinomyces (Actinobacteria) | 1.60% | Unclassified, Family Clostridiaceae | 1.46% |
| **Mouth end bloat scrape** | | **Stomach end bloat scrape** | |
| Unclassified, Family Planococcaceae | 31.8% | Morganella (Proteobacteria) | 85.8% |
| Ignatzschineria (Proteobacteria) | 26.0% | Unclassified | 6.98% |
| Unclassified, Family Clostridiaceae | 11.1% | Unclassified Bacteria | 2.30% |
| Unclassified, Order Bacillales | 8.64% | Eggerthella (Firmicutes) | 1.32% |
| Unclassified, Family Clostridiaceae | 4.86% | Unclassified, Family Carnobacteriaceae | 0.911% |
| Unclassified, Family Carnobacteriaceae | 4.32% | Sphingomonas (Proteobacteria) | 0.597 |
| Erysipelothrix (Firmicutes) | 2.92% | Unclassified, Family Coprobacillaceae | 0.440% |
| Unclassified, Phylum Firmicutes | 2.39% | Propionibacterium (Actinobacteria) | 0.314% |
| Vagococcus (Firmicutes) | 1.93% | Vagococcus (Firmicutes) | 0.251% |
| Unclassified, Order Bacilli | 1.30% | Unclassified, Order Rickettsiales | 0.220% |
| **Small intestine end bloat scrape** | | **Transverse colon end bloat swab** | |
| Unclassified | 37.1% | Unclassified, Family Coprobacillaceae | 24.5% |
| Unclassified, Family Clostridiaceae | 26.3% | Unclassified | 22.8% |
| Unclassified Bacteria | 13.1% | Eggerthella (Firmicutes) | 20.2% |
| Unclassified, Family Peptostreptococcaceae | 6.30% | Eubacterium (Firmicutes) | 3.27% |
| Faecalibacterium (Firmicutes) | 4.30% | Escherichia (Proteobacteria) | 3.03% |
| Klebsiella (Proteobacteria) | 2.29% | Parabacteroides (Bacteroidetes) | 2.07% |
| Collinsella (Firmicutes) | 1.15% | Ruminococcus (Firmicutes) | 1.87% |
| Unclassified, Family Ruminococcaceae | 0.973% | Bifidobacterium (Actinobacteria) | 1.71% |
| Clostridium (Firmicutes) | 0.801% | Enterococcus (Firmicutes) | 1.59% |
| Streptococcus (Firmicutes) | 0.744% | Unclassified, Family Ruminococcaceae | 1.47% |
| **Fecal scrape** | | **Fecal Scrape** | |
| Unclassified, Family Ruminococcaceae | 23.9% | Unclassified, Family Peptostreptococcaceae | 22.4% |
| Faecalibacterium (Firmicutes) | 12.6% | Unclassified, Family Ruminococcaceae | 9.81% |
| Unclassified, Ruminococcaceae | 12.3% | Collinsella (Firmicutes) | 7.18% |
| Eubacterium (Firmicutes) | 10.0% | Klebsiella (Proteobacteria) | 6.51% |
| Collinsella (Firmicutes) | 6.74% | Faecalibacterium (Firmicutes) | 4.57% |
| Ruminococcus (Firmicutes) | 4.44% | Turicibacter (Firmicutes) | 4.47% |
| Ruminococcus (Firmicutes) | 3.74% | Megasphaera (Firmicutes) | 4.22% |
| Unclassified, Family Peptostreptococcaceae | 2.99% | Unclassified, Family Ruminococcaceae | 4.09% |
| Unclassified, Family Lachnospiraceae | 2.83% | Eubacterium (Firmicutes) | 3.18% |
| Unclassified, Class Clostridia | 2.73% | Unclassified, Family Clostridiaceae | 2.96% |

**Supplementary Table 3:** Relative abundances of the top ten genera present in each sample from body 016.
